# Climate emotions and climate justice: exploring the role of affective engagement and existential experience in sport for development and peace

**DOI:** 10.3389/fspor.2026.1678036

**Published:** 2026-07-06

**Authors:** Christina T. Kwauk, Lyndsay M. C. Hayhurst, Isra Iqbal

**Affiliations:** 1 Unbounded Alliance, University Park, MD, United States; 2 York University School of Kinesiology and Health Science, Toronto, ON, Canada; 3 Western University School of Kinesiology, London, ON, Canada

**Keywords:** affective pedagogies, climate action, climate change, climate change education, climate emotions, climate justice, outdoor adventure sports and recreation, sport for development

## Abstract

Global policy discourse increasingly frames sport, leisure, and recreation as tools for advancing climate justice alongside education, health, and social cohesion. While initiatives such as the United Nations Sports for Climate Action Framework highlight sport's potential to address the climate crisis, little is known about how SDP can foster meaningful climate action – defined as actions that contribute to transformative and equitable climate solutions that produce measurable greenhouse gas emissions reductions and support systemic change, especially in the face of climate vulnerabilities structured by intersecting inequalities of class, race, gender, and postcoloniality (IPCC, 2023). Indeed, research on the knowledge-to-action gap in climate change education underscores the importance of affective experiences and existential reflection as mediators between environmental learning and action. In this paper, we argue that physical culture – especially the use of outdoor adventure sports and recreation (OASR) – when deployed in the context of SDP, is *potentially* uniquely positioned to cultivate environmental identity through embodied, emotional engagement with nature. We further contend that – because SDP initiatives operate in social domains directly impacted by climate change – they are well positioned to advance climate justice. In this perspectives piece, we look across disciplines to explore the role of affective engagement and existential experience in sport as a pedagogical entry point for climate action, climate activism and climate justice. We conclude that SDP offers an unrealized space for socioemotional and behavioural learning – often neglected in climate change education – that can strengthen pathways from climate knowledge to climate action for climate justice.

## 1 Introduction

Sport for development and peace (SDP) positions sport, leisure, recreation and play-based interventions to positively contribute towards non-sport goals related to education, health, gender, livelihoods, disability, peace, social cohesion and, most recently, to climate justice (1, 2). Here, we refer to climate justice as both the equitable outcomes and the processes through which societies identify and address the ways the climate crisis reflects and exacerbates interconnected patterns of social injustice, especially as they pertain to the disproportionate distribution of its burdens and potential solutions (3, 4). The United Nations Sports for Climate Action Framework, for example, calls upon SDP initiatives to urgently address the climate crisis raising awareness about climate change – or the changes in the Earth's climate caused directly or indirectly by human activity (5) – and to advocate for climate action, especially as some sports are directly being impacted by the impacts of climate change (6). Climate action includes substantive and sustained actions that reduce greenhouse gas emissions, strengthen resilience to climate impacts, and support systemic change rather than superficial or symbolic responses (7). Climate activism extends such action to raising awareness and educating and engaging others to address the effects of climate change (8). Surprisingly, we still know very little about how sport, leisure, recreation and play – especially in the context of SDP – *may* generate climate action and meaningful engagement in transformative and equitable climate solutions that not only reduce emissions but also promote intersectional climate justice (9, 10).^1^

Research on the knowledge-to-action gap, explored often by scholars of climate change and environmental education, environmental behavioral economics, and environmental psychologists, points increasingly to the role of affective experiences and emotional engagement that invites existential reflection on issues of climate change and climate justice as important mediators between knowing and doing (11–14). Although the nexus between SDP and climate action remains largely understudied, we suggest that physical culture, and especially the use of outdoor adventure sports and recreation (OASR) when deployed in the context of SDP, is *potentially* distinctively positioned to facilitate or strengthen the environmental identities of participants through both affective and existential experiences.^2^

In this paper, we argue that there is an opportunity to work in/through SDP to cultivate and understand the role of climate emotions in mediating environmental learning-to-climate-action (here conceptualized as driven by a ‘what can I do?’ attitude) to climate activism (driven by a ‘what can we do, together?’ mindset), especially to advance climate justice for communities and populations unfairly burdened by the impacts of climate change. We suggest this is critically important because emotional engagement – and the existential experience it creates – is a pedagogical phenomenon that remains understudied. Better understanding this process could support its intentional cultivation within SDP programs and its integration into climate change and environmental education as a critical missing link in achieving transformative learning outcomes. As highlighted by a notable body of evidence, environmental and climate learning is heavily biased toward cognitive knowledge; the socioemotional and behavioural domains are grossly neglected (16, 17). Here, SDP offers an important space to pursue questions pertaining to the role of affect in mediating existential experiences – experiences that challenge one's ways of thinking, being, and doing – and the development of environmental identity that is critical to facilitating climate activism, collective action, and climate justice (18–20). At the same time – and importantly – we recognize that the lived, affective, and existential dimensions of SDP are contextually situated and geopolitically contingent. Accordingly, we would be remiss not to acknowledge the dangers involved in assuming a homogeneous ‘environmental identity’ that OASR would monolithically ‘activate’ to encourage PEBs.^3^

To cement these arguments, we put recent literatures anchored in sport, physical culture and the environment [e.g. (21, 22, 74)] in conversation with scholarship focused on affect in climate change education^4^ and climate justice education^5^ [e.g. (12, 23, 24); Ray, 2020] the role of emotions like hope, fear, anger, humour, and anxiety in climate activism [e.g. (27–30, 75)], to urge scholars to explore the nexus of climate emotions, existential reflection, and environmental identity formation in SDP. Though recent scholars have documented how sport and recreation have far-reaching negative impacts on the environment, including overconsumption, landscape degradation, ecosystem disruption and pesticide usage (74, 76); it is also important to note how the sport and leisure sectors have, at the same time, facilitated pro-environmental behaviors (PEB) and can be more intentionally leveraged as a pedagogical tool to advance climate action and climate justice.

## 2 Sport for climate action and justice: pathways, practices, and possibilities

### 2.1 Confronting climate change through emotional connection and Pro-environmental behaviors: opportunities to bridge SDP to climate action

Communities targeted by SDP initiatives are often located in contexts experiencing high or extreme vulnerability to the impacts of climate change with little to no access to adaptation and resilience resources (1, 31). Scholars such as Knowles et al. (32) and Giulianotti et al. (1) suggest that SDP NGOs may provide an effective platform through which to promote climate action and climate justice by using sport to address social inequities in education, gender, and health that shape underlying climate vulnerabilities of individuals and communities (33–35). As such, SDP NGOs could be well positioned to advance climate action and to promote climate justice even when environmental objectives are not explicit program goals. Nonetheless, there remains an opportunity to better understand precisely *how* SDP can *more directly* advance climate action as a vehicle for climate and environmental learning in itself.

Indeed, emerging research highlights how physical culture can ignite climate action through climate activism (36–38). But what is it that facilitates or sparks that activism? Elite and professional athletes, such as Brent Suter and Chloe Kim, and grassroots NGOs, such as Protect Our Winters, We Play Green, and Surfers Against Sewage, have been at the forefront of climate activism in sport (21, 32, 33, 38, 39). These athletes and organizations not only lean on the urgent need to ensure present and future generations can enjoy outdoor sports threatened by climate change. Crucially – and relevant to this paper – they *also* draw on a shared emotional connection with nature made possible by their sports to mobilize individuals to protect the environment and to stop climate change through collective climate action.^6^ It is precisely *this* capacity of sport and physical culture – especially OASR – to deepen emotional connections with nature and the environment, that underpins this paper's exploration into the propensity of SDP to serve as an important space for environmental and climate learning to promote pro-environmental behaviours (PEBs) necessary for climate action (41, 42).

PEBs can be described as “behaviours undertaken by a single individual to reduce one's negative environmental impact with a clear intention to change the environment” [77, p. 2; see also (78)]. However, the role of emotions in sport and in SDP initiatives, and their promotion of PEBs and climate activism, remains an underexplored area in scholarly research. Critically, promoting PEBs is not simply a matter of linear progression from education (or sport, in this case) to action as early rationalist models suggested (79). That is, increased environmental knowledge does not automatically lead to environmentally responsible behaviour; rather, pro-environmental action is shaped by a combination of many individual and structural factors, including a sense of environmental identity, connection to nature, and perceived self-efficacy (20, 43–45). And yet, this begs the question of *how,* then, might SDP, and in particular the emotional connection to nature potentially offered by OARS, act as a conduit for climate learning and facilitate PEBs, climate activism, and climate action?

### 2.2 Green design elements for transformative climate learning: A “green” pedagogical framework for SDP?

**Figure 1:**
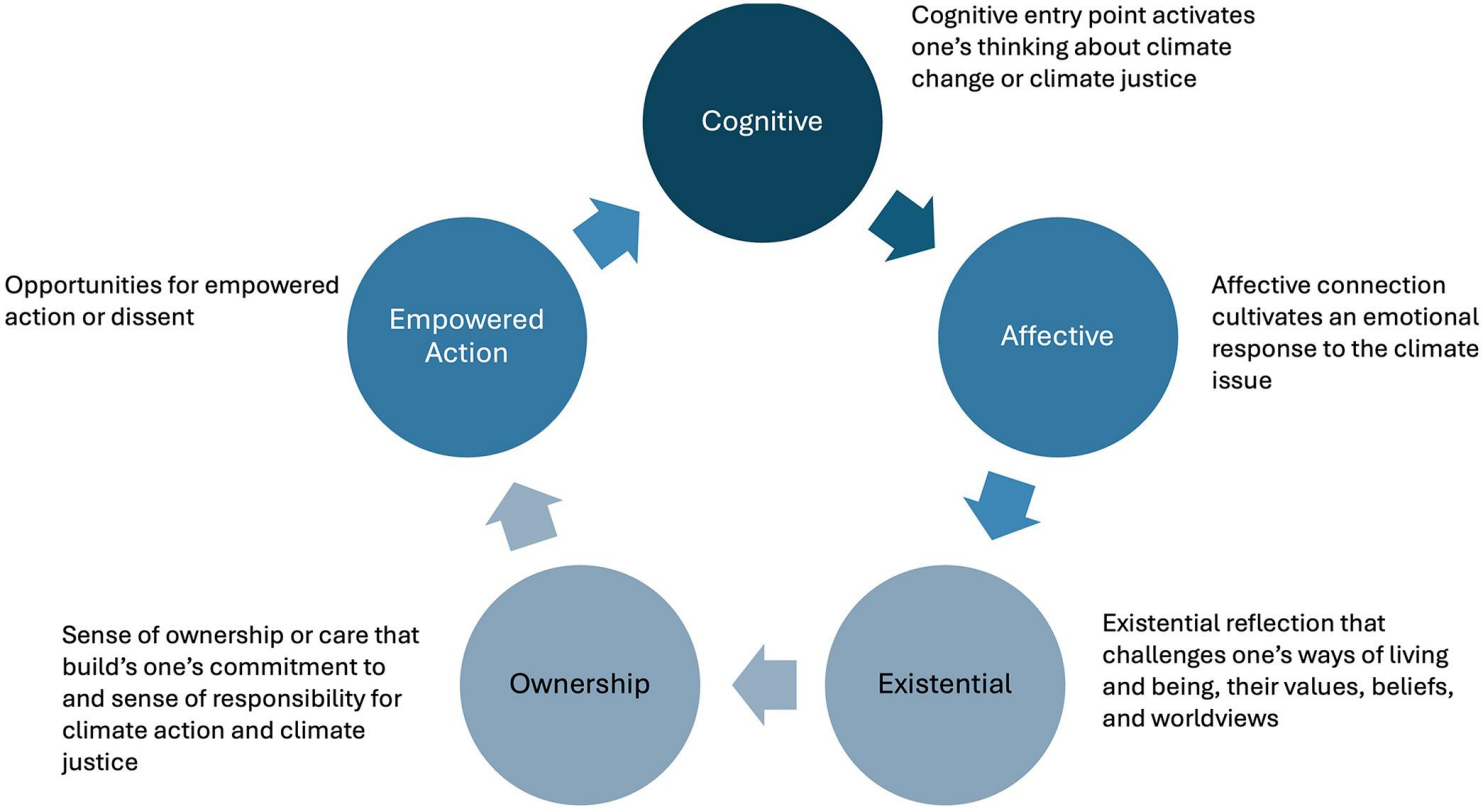
Green design elements for transformative climate learning. Adapted from Kwauk and Casey (18).

The question as to what shapes PEBs is complex and cannot be answered in a simple causal framework (78). Recent SDP literature, for example, highlights the importance of a socio-ecological approach that reconceptualizes the SDP sector as an ecological endeavor in and of itself, instead of a tool through which to simply ’spread the news’ (37). In turn, research in climate change and environmental education suggests an iterative and holistic process with several “key ingredients” or programmatic design elements can help facilitate the translation of knowledge into action (11, 46). These include: (i) a **cognitive point of entry** that activates one's thinking about climate change, like news about a current climate-related disaster or a breakthrough in a climate solution; (ii) an **affective connection** that cultivates an emotional response toward the environment or to the people or the more than human world impacted by the climate event; (iii) an **existential experience** that challenges one's sense of self, one's way of living and being, one's values, beliefs and worldviews by introducing new ideas, concepts, realities, and/or perspectives; (iv) a sense of **ownership or care** for others that builds one's personal connection and/or commitment to and sense of responsibility for or identification with climate action and climate justice; and (v) opportunities for **empowered action or dissent**, increasing one's sense of agency, confidence, and direction (18) (see Figure 1).

These green design elements draw on and align with scholarship on experiential and transformative learning and critical pedagogy – fields that, we argue, SDP could engage more intentionally to translate knowledge into action [see (47–49, 80)]. Indeed, SDP provides a unique opportunity for affective connection through embodied learning experiences, where participants may experience diverse and sometimes conflicting climate emotions of hope and optimism together with intense feelings of anger, climate anxiety, despair, eco-grief, exhaustion, fear, guilt, and solastalgia (23, 50, 51, 81). Despite these “negative” emotions that SDP experiences may stir up in participants, Bechtoldt and Schermelleh-Engel (52) and others (11, 19, 23) note that climate-related worry can motivate activism and encourage individuals to take action more than positive emotions (13, 75, 82). This is especially the case if such affective and existential experiences are paired with structured reflection or guided opportunities for empowered action and the development of a personal sense of ownership of or care for environmental and climate justice issues (18). In short, we argue that SDP programs - especially those leveraging OASR – have a distinctive pedagogical entry point for transformative climate learning by creating opportunities to forge affective connections with nature and to engage in existential reflections on our relationship with the environment.

### 2.3 OASR as an SDP conduit for affective experiences and existential reflection

Indeed, individuals involved in OASR may be more likely to engage in PEBs, as such activities can activate both pro-social motivations (e.g., concern for collective ecological well-being) and relevant egocentric psycho-social factors (e.g., personal responsibility or feelings of guilt) (53, 54). These overlapping motivations suggest that engagement with nature through OASR can simultaneously cultivate individual and societal commitments to environmental action. Existing research has shown that OASR activities can aid in facilitating PEBs through holistic learning opportunities because they take place in the natural world and are “largely self-regulating and allow for a (sometimes communal) celebration of play, creativity and self-expression” (55, p. 3). Examples of OASR activities include climbing, canoeing, mountain or bush walking, skiing, biking, surfing, fishing, hiking and caving. The very nature of these activities positions them as a medium for the development of a deeper environmental identity and recreational activism (83), though we acknowledge that their relevance across diverse SDP contexts—shaped by geography, culture, and socioeconomic access—remains an important area for future research. An environmental identity can be defined as, “extend[ing] beyond this anthropocentric idea of an “I” that is other or opposed to the natural world and instead encompass[ing] a sense of self that is part of the natural world” (42, p. 330). And yet, there remains a disconnect when using traditional sports (i.e., football, basketball, ice hockey) to instill an environmental identity, likely *because these activities only provide opportunities to learn about the environment rather than experience emotional connection and immersion in the environment* [see (55)].

In essence, developing an environmental identity – specifically, fostering a strong emotional connection to nature – is a step toward acting in environmentally responsible ways to help preserve the natural world (20, 84). While participation in some sports can foster an emotional bond to the environment, engagement with OASR promises to be an engaging pathway to build one's environmental identity (42, 56) and thus their personal commitment to care for others, including the environment, and to engage in climate action. Particularly, individuals who regularly engage in outdoor recreation activities are more likely to adopt pro-environmental behaviours (57–58). However, simply taking part in OASR initiatives does not automatically make a person more likely to engage in PEBs; instead, their emotions, values, attitudes, and awareness play a crucial role in acting pro-environmentally [see (56)]. And we argue that it is through the *intentional* design of SDP initiatives that such embodied, affective experiences outdoors can create opportunities for existential reflections that – when coupled with opportunities for ownership and care – mediate empowered climate action. Intentionality here is key: without the intentional integration of these green design elements, SDP programs—with or without OASR components—can inadvertently perpetuate harmful environmental practices and behaviors—for instance through the production of environmental waste and pollutants, through the consumption of sporting goods and equipment with high carbon footprints, through the alienation of Indigenous populations from the use of traditional lands for recreation.

### 2.4 Taking the leap to climate justice

At this juncture it seems important to critically question whether and how SDP can truly advance climate justice without shifting the burden of climate action and climate activism onto the very communities – often in the Global South, and especially women, girls, and Indigenous peoples – who have contributed least to the crisis yet face its harshest impacts. Indeed, given the disproportionate burden of climate impacts borne by women and girls around the world (59), we highlight the gendered dimensions of climate emotions and the emotional labor borne by women and girls and athlete activists carrying out global environmental activism, especially in contexts where climate stress exacerbates gender inequality and gender-based violence (60–62). We contend that ‘intersectional environmentalism’ [cf. (10),] is a useful lens for understanding how diverse experiences of nature shaped by the intersecting identities of ‘athlete environmentalists’ can go on to shape diverse environmental identities further etched by gender, race, and class [cf (63, 64, 73)]. Such an understanding of intersectional environmentalism could help inform the ways in which SDP engages with the transformative potential of embodied climate and environmental learning, strengthening individuals’ self-awareness, sense of resistance, understanding of power, and their capacity to build partnerships, coalitions and relationships of reciprocity and solidarity—examples of the transformative skills needed to more boldly translate knowledge into empowered action that contributes to climate justice (65, 66).

As such, understanding climate emotions and PEBs in SDP must be understood in a broader geopolitical and historical context and through local and lived experiences of the climate crisis [see (67); see also (68)]. The negative impacts of climate change, particularly for countries in the Global South, originate from centuries of colonial and capitalist exploitation that have left these countries with limited resources to address adverse climate effects [see (69)]. Engaging in climate activism in the Global South is thus also a fight against the enduring legacy of colonialism (70). Making the leap from climate activism to climate justice thus means moving beyond individual behaviours to collective action and structural change.^7^ Such a move is essential to avoid reinforcing neoliberal logics and to instead leverage SDP to transform the systemic inequalities, unequal relations of power, and unfair distribution of resources driving climate injustice.

Critically, this means SDP and SDP scholarship has a responsibility to explore how intersectional environmentalism—i.e., the recognition that environmental harms intersect with social inequalities structured by race, gender, socioeconomic status, and other systems of disadvantage (10).—can be integrated into programming as a cognitive entry point (one of the five green design elements discussed earlier) for participants to explore climate solutions that address interlocking social and environmental drivers of climate vulnerability and structural inequality (71). Here, SDP NGOs must approach climate justice in a way that is co-created with those communities most impacted. Importantly, this must be done in a way that is sensitive to – and conscious of – the risk of political violence experienced globally by environmental defenders.

## 3 Discussion

Climate emotions and the existential experiences they evoke are key ingredients to the translation of climate knowledge into climate action. But the education sector, and especially the field of climate change education, has not quite figured out how to leverage this pedagogically or as program design elements. Research on climate colonialism and on climate justice education reminds us to recognize the differential emotional toll of climate change and to foster climate resilience, critical thinking, and action in/through programming, practices and policies (23, 70, 72).

Meanwhile, the SDP sector offers fertile ground for exploring and understanding how sport's affective engagement and embodied learning can help to bridge knowledge and awareness about the urgency of the climate crisis to the promotion of climate action, activism, and climate justice. OARS in particular appears to have all the central components of a well-designed climate change education program that the broader SDP sector (as well as the education sector) could learn from, including: 1) the affective connection to nature; 2) the existential reflection this invokes that creates an acute awareness of the impacts of climate change; coupled with 3) a sense of ownership and care for the environment and the opportunity to engage in empowered action (18). If nestled within an intersectional approach to environmentalism, SDP, sport, and physical culture more broadly can play a critical role promoting climate activism and climate justice.

We urge researchers, educators, and practitioners to begin exploring the roles of emotion and embodiment in the promotion of climate activism and climate justice through SDP, and sport more broadly. Further, more research is needed to: 1) conceptually distinguish between SDP practices that foster environmental awareness and those that do not; and 2) more deeply engage in transformative learning, critical pedagogy, and climate justice education to help SDP more intentionally advance climate activism toward climate justice.^8^ We understand intuitively from the confluence of different fields of literature that SDP must fully engage place-based learning, embrace its emotional connection to nature, seek out locally-attuned voices, recognize pre-existing social inequalities, motivate a vision for the future, and establish practical solutions (33, 37). But for SDP to become a platform for climate justice, we must better understand how to mediate contextually relevant and deeply personal emotional experiences with nature and how to guide contextually relevant existential reflections that help strengthen an environmental identity for climate activism and the promotion of climate justice.

## Data Availability

The original contributions presented in the study are included in the article/Supplementary Material, further inquiries can be directed to the corresponding author.

## Footnotes

^1^ Of course, we acknowledge that different activities also have pertinent histories of environmental harm, exploitation and damage (e.g., golf, skiing, etc.). Thus, while OASR activities hold the potential to contribute to the development of environmental identities, these are not monolithically ‘positive’ nor homogeneously taken up as such. We thank an anonymous reviewer for pointing this out.

^2^ We acknowledge that OASR is historically associated with individualized, high middle-class sports disciplines and that their use in SDP and alignment with the lived contexts of many SDP participants remains an open and important question [see (15)]. Empirical data on the prevalence of OASR within SDP programming is currently limited, and we recognize the tensions between these fields deserve further scholarly attention. Recent research by Yerashotis et al. (2025) (15) notes that, “Outdoor education […] may […] benefit from forming organizational relationships with the SfD sector, as the latter has proven more adept at engaging with marginalized groups, and especially disadvantaged youth, than the former,” (p. 443). Within the scope of this paper, however, we position OASR not as a universal or uncritical model for SDP, but as one potential pedagogical entry point or example—particularly where geographic, cultural, and institutional conditions make such activities accessible—for cultivating climate emotions and bridging environmental learning with climate action.

^3^ Thank you to an anonymous reviewer for pointing this out.

^4^ Climate change education helps learners understand the causes, impacts, and solutions to climate change, and equips them with the knowledge, skills, values, and attitudes to respond effectively.

^5^ Climate justice education extends the scope of climate change education by helping learners understand how climate change is interconnected with issues of inequality and human rights, equipping learners with the knowledge, skills, values, and attitudes to take ethical action toward fair and inclusive climate solutions that center the experiences, knowledge, and leadership of communities most affected by climate change, including Indigenous peoples, low-income communities, and countries in the Global South. Climate justice education explores the historical, political, and economic drivers of the unequal distribution of the burden of impact and the unequal access to resources and finance to adapt to these impacts (25, 26).

^6^ Collective climate action is where individuals come together as members of a group to change the socio-political structures and socio-economic systems driving climate change (40)

^7^ It is through collective action that individuals come together as members of a group to change the socio-political structures and socio-economic systems driving climate change (40).

^8^ Thank you to two anonymous reviewers for suggesting these avenues for future research.
